# Malaria in the Australian Military, 2008–2022

**Published:** 2024-08-20

**Authors:** Christopher Moller, Ken Lilley, Fiona McCallum, G. Dennis Shanks

**Affiliations:** 1 Australian Defence Force Malaria and Infectious Disease Institute, Enoggera, Queensland; 2 University of the Sunshine Coast, Queensland, Australia; 3 University of Queensland, School of Public Health, Brisbane, Australia

## Abstract

Australian Defence Force (ADF) personnel train and operate in malarious regions that include neighboring countries with high burden and species with latent hepatic parasites.^1^ We summarized longitudinal malaria case data, following a prior 10-year period review to 2007.^2^ Malaria case entries within the ADF Malaria and Infectious Diseases Institute (ADFMIDI)-managed Central Malaria Register (CMR) were examined. Data from cases confirmed between January 1, 2008 through December 31, 2022 were analyzed. Sixty ADF members were diagnosed with malaria, including 1 with a mixed *Plasmodium falciparum* and *P. vivax* infection. Of 61 malaria infections, 69% (42 of 61) were *P. vivax*. *P. vivax* infection resulted in delayed initial case presentation (more than 4 weeks after exposure) in at least 36% (15 of 42) of cases, and 5 personnel experienced further relapse. Most *P. vivax* infections were acquired in the U.S. Indo-Pacific Command (INDOPACOM) and *P. falciparum* in the U.S. Africa Command (AFRICOM) regions. The ADF experienced ongoing reduced malaria case incidence following high rates in the early 2000s. Maintenance of prophylactic vigilance, both for eradicating dormant hypnozoites and preventing *P. vivax* relapse, remains important, however.

**What are the new findings?:**

Between 2008 and 2022 annual malaria case rates among Australian military personnel were approximately 5.4 per 100,000 person-years, or one-third of Australian civilian case rates. Most cases were caused by *P. vivax*, acquired in Papua New Guinea within the U.S. Indo-Pacific Command, which reflects high regional endemicity and the regular training and operations within the region.

**What is the impact on readiness and force health protection?:**

Malaria rates among regionally-deployed personnel remain relatively low following high rates, and medical response, in the early 2000s. Ongoing *P. vivax* predominance and relapse, however, highlight that vector bite prevention awareness, adherence to drug regimens, as well as efficacious new anti-malarials to improve prophylaxis and combat dormant hypnozoite forms, remain necessary.

## 1 BACKGROUND

Australia is within the U.S. Indo-Pacific Command (INDOPACOM) Area of Operations. While malaria is no longer endemic to Australia, deployments in the region have resulted in significant numbers of active troops being infected,^2^ with risk of loss of combat manpower from illness. Many near neighbors have high burdens, with a high proportion of cases caused by hypnozoite-forming *P. vivax* and *P. ovale*, compared to other World Health Organization (WHO) regions.^1^ This presents unique challenges for malaria prevention and surveillance due to increased risk of delayed presentation and relapse.^3,4^ There are no diagnostic means to determine hypnozoite carriage,^5^ and a limited number of medications available to clear hypnozoites from the liver,^6^ making force health protection in INDOPACOM challenging.

Risk-based Australian Defence Force (ADF) health guidance against malaria mandates the use of personal protective measures, including long-sleeved and -legged uniforms, bed nets, permethrin treatment of uniforms and nets, DEET-based topical repellent and compliance with chemoprophylaxis regimens. Doxycycline has been the first-line chemoprophylaxis against malaria since 1990. On leaving a malarious area, primaquine is used for eradication and radical cure in personnel without glucose-6-phosphate dehydrogenase deficiency. Alternative prophylactic regimens using atovaquone/proguanil, since 2006, or tafenoquine, registered in 2018, are available for those members in whom this protocol is not suitable. Military members presenting with fever within 3 months of deployment to a malarious area are tested for malaria, including peripheral blood polymerase chain reaction (PCR).

The ADF Malaria and Infectious Disease Institute (ADFMIDI) conducts surveillance and research to protect personnel against malaria and other vector-borne diseases. ADFMIDI maintains the Central Malaria Register (CMR), which receives and collates demographic, travel, disease, and treatment information from suspected and confirmed cases of malaria in ADF personnel. CMR enables disease trend monitoring, reporting, and response within the ADF. The most recent published review of malaria cases in the ADF covered the 10-year period preceding and including 2007.^2^ It documents massive *P. vivax* burden following large-scale personnel deployments in the Southwest Pacific region, with outcome modifications to primaquine regimen, vector control, and personnel preventative awareness. *P. vivax* relapse may be associated with reduced primaquine metabolism.^7,8^ Factors other than human cytochrome P450 2D6 (CYP2D6) phenotype, however, such as personnel adherence or parasite tolerance to primaquine, were found likely to contribute.^9^ This report describes confirmed malaria cases reported to the CMR database during the 15-year period from 2008 to 2022.

## 2 METHODS

The ADF maintains a register of personnel diagnosed with malaria. Case reporting by diagnosing clinician or health center to ADFMIDI, via template electronic form, is mandatory under the direction of ADF Joint Health Command. CMR database information includes patient demographic details, military service type, illness onset date, malaria diagnosis date, malaria species, dates of movement between countries, country of acquisition, and administered prophylaxis and eradication treatments.

All 90 ADF CMR entries from January 1, 2008 through December 31, 2022 were exported from the database and individually cross-checked against original reports received by ADFMIDI. Suspected reported cases without confirmatory microscopy- or PCR- pathology data were excluded. Average ADF malaria case rates for the 15-year time period were determined using 2015 (mid-year) demographic data, for which both age group and gender data were available, as denominator.^10^ Comparison Australian figures were sourced and determined using the Australian Government National Notifiable Disease Surveillance System data.^11^ ADF malaria cases occurred either ‘during travel’, if disease onset was prior to departure from a malarious area, or ‘after travel’, if disease onset occurred after leaving the malarious area. Malaria cases of *P. vivax* and *P. falciparum* were classified as ‘delayed’ if clinical presentation was more than 28 days after departure from a malaria-endemic area.^12^ *P. vivax* disease case presentation occurring subsequent to primary clinical presentation, with no chance for malaria re-exposure, was defined as ‘relapse’ and was not counted as a separate infection. Case demographic information was summarized. Countries of malaria acquisition were grouped within unified combatant command regions for result analysis and presentation.

## 3 RESULTS

During the 15 years from January 1, 2008 through December 31, 2022, 60 Australian military members were diagnosed with malaria. One member had a mixed species infection consisting of *P. vivax* and *P. falciparum *acquired from, and presenting in, Sudan—in the U.S. Africa Command (AFRICOM) region—to total 61 infections in 60 personnel, representing an annual incidence rate of approximately 5.4 cases per 100,000 person-years (p-yrs) among ADF personnel (**Table**). The majority of malaria infections were *P. vivax* (42/61, 68.9%, 3.7 cases/100,000 p-yrs), followed by *P. falciparum* (13/61, 19.7%, 1.1 cases/100,000 p-yrs). Three cases were caused by unknown malaria species, while *P. knowlesi*,^13^ *P. malariae*, and *P. ovale* caused 1 infection each. Infections occurred most commonly in Regular Forces personnel (59/61, 96.7%, 5.2 cases/100,000 p-yrs), Army members (50/61, 82.0%, 4.4 cases/100,000 p-yrs), and male service members (57/61, 93.4%, 5 cases/100,000 p-yrs). The age group most infected was 20-29 years (29/61, 47.5%, 2.6 cases/100,000 p-yrs).

**Figure 1:**
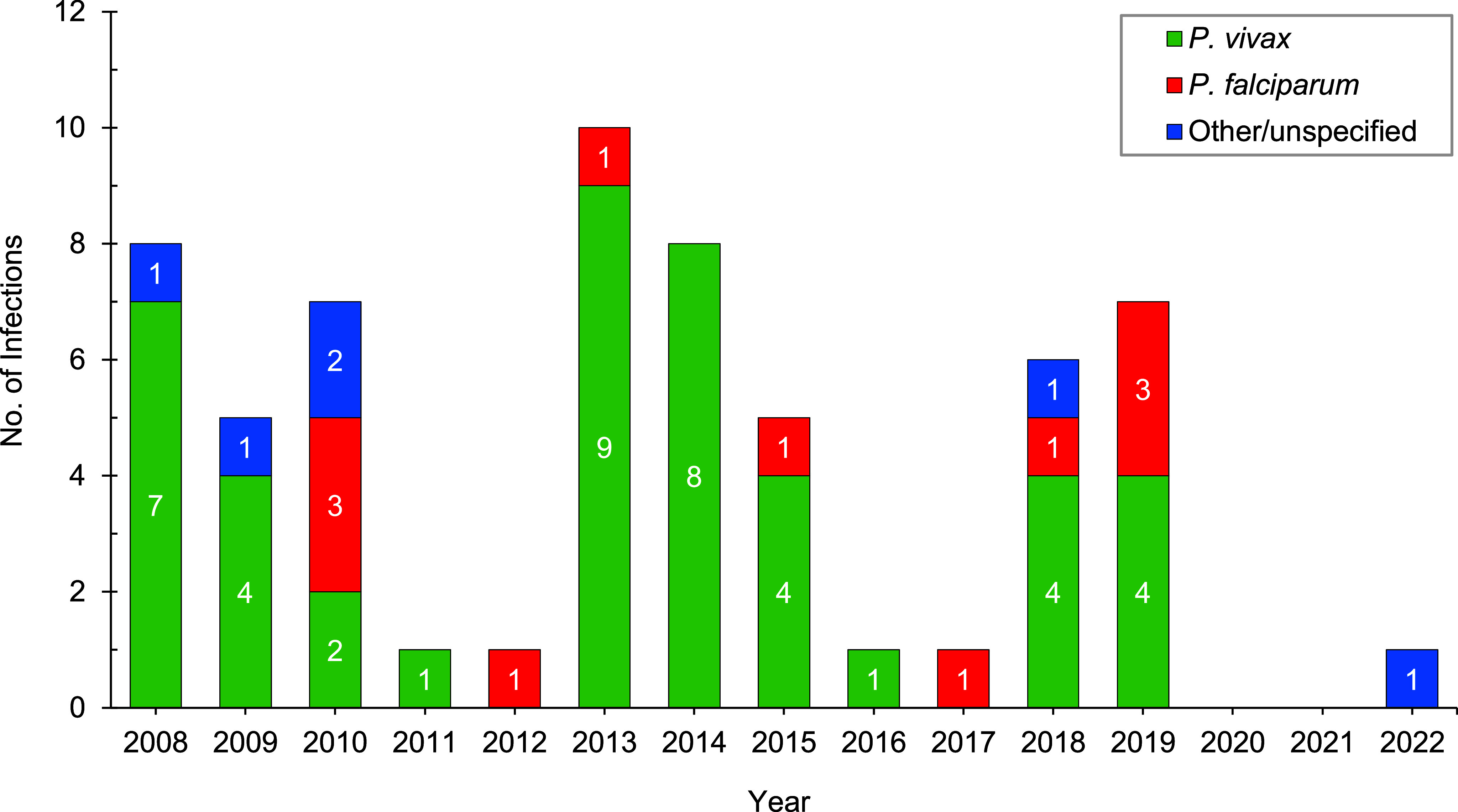
Malaria Infections in ADF Personnel by Year, 2008–2022

**Figure 2:**
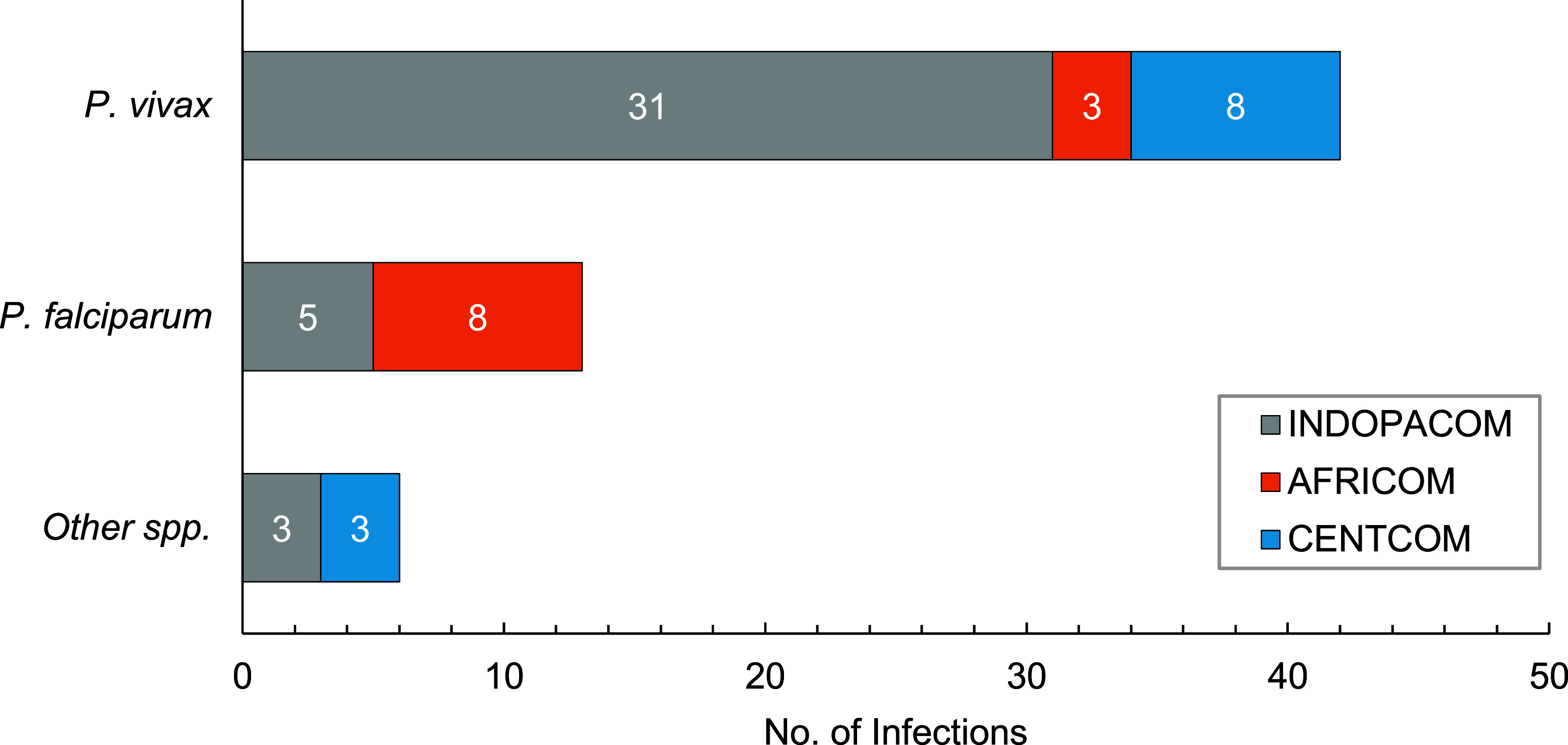
Malaria Infections by Species and Region of Acquisition, ADF Personnel, 2008–2022

**Supplementary Figure 1:**
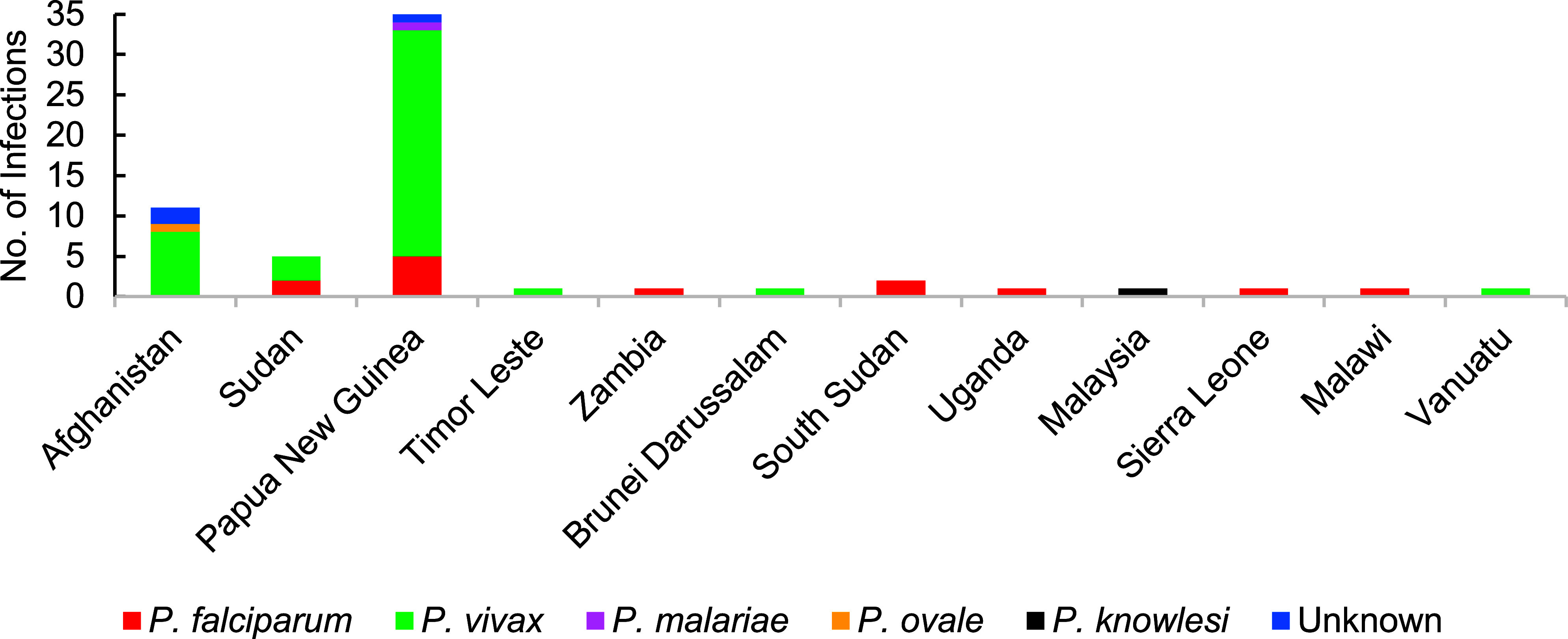
Number of Malaria Infections in ADF Personnel by Species and Country of Acquisition

At least 1 confirmed malaria case (1.3/100,000 p-yrs) occurred annually during most years of the 15-year period under review (**Figure 1**). The frequency of malaria case presentation was variable across the time period, including a notable absence of cases in 2020-2021, coinciding with a reduction in all travel and deployment activities due to the COVID-19 pandemic. Most malaria infections were acquired in the INDOPACOM region (39/61, 63.9%, 3.4/100,000 p-yrs), including 35 from Papua New Guinea. Eleven infections (11/61, 18.0%, 0.9/100,000 p-yrs) each also occurred from the AFRICOM and CENTCOM regions (**Figure 2**, **Supplementary Figure 1**). *P. vivax* in members returned from the INDOPACOM region made up half of the total infection count (31/61, 50.8%), while the majority of *P. falciparum* infections were from the AFRICOM region (8/13, 61.5%), consistent with the most common circulating malaria species.

Complete entry and exit dates to malarious areas were available for 53 of the 60 personnel, with 20 cases presenting during travel and 33 presenting after travel. Case onset during travel did not appear to follow any temporal pattern (**Supplementary Figure 2a**). Both *P. falciparum* and *P. vivax* infections occurred in personnel who presented after travel (**Supplementary Figure 2b**). Fifteen personnel had delayed case presentation caused by *P. vivax*, likely due to parasite re-emergence from liver hypnozoites. Of the 42 personnel who experienced *P. vivax* infection, 5 (5/42, 11.9%) experienced relapse, including 3 individuals who reported partial or complete non-compliance with the primaquine eradication regimen.

Time of onset to first relapse following the start of *P. vivax* case treatment was 43 to 210 days (median 67 days). One of these 5 people went on to have a second relapse 69 days after commencement of a known-completed course of primaquine, prescribed following the first episode of relapse.

## 4 DISCUSSION

Australian military forces commonly undertake exercises, assistance operations, and joint military activities with other host countries in INDOPACOM. The determined annual malaria case rate of 5.4 per 100,000 p-yrs in ADF personnel is higher than that experienced by U.S. Armed Forces, which reported 403 cases during the 10-year period 2012 to 2021, and a rate of 1.4 per 100,000 p-yrs following 30 infections recorded during 2022.^14,15^ This difference in case rates may reflect the closer proximity of Australia to malaria-endemic countries in addition to the dislocation of U.S. service members’ duty locations from the regions of malaria transmission within their malaria-endemic countries of deployment.^15^ Differing force chemoprophylaxis approaches, including drug mechanism and site of action, may also play a role.^16^ This ADF malaria case rate is approximately one-third of that determined from the Australian civilian population over the same period (15.8/100,000 p-yrs). A lower awareness or uptake of malaria preventative and prophylactic measures by civilian travelers compared with ADF personnel, and differing regions of travel, may contribute.

The finding that ADF malaria infections occurred most commonly in young adult men in full-time service with the Australian Regular Army broadly reflects the demographics of the Australian military.^10^ The high malaria infection rate of 4.4 cases per 100,000 p-yrs within the Army compared with the other 2 services likely reflects an increased chance of malaria exposure resulting from the typical environments and activities of land-based forces, compared to those of air or sea operations, as well as the higher number of Army compared with Air Force or Navy personnel in the ADF. The distribution of infections between sexes was disproportionate to the demographics of the ADF, with only 6.6% (4/61) occurring in female service members compared to a reported ADF female participation rate of 15.1%.^10^ The reasons for this are unclear based on the data available, but reflect the possibility for reduced female, compared with male, force deployment ratios to malaria-endemic regions.

Annual ADF malaria case numbers, though relatively low, differed markedly during this 15-year period. No major changes in malaria prevention or prophylactic drug or dose regimens, to which this difference could be attributed, were adopted over this time. The majority of cases occurred from Papua New Guinea, including clustered outbreaks in 2013-2015 and 2018-2019, following conduct of annual training exercises in country regions with high rates of malaria transmission. Additionally, intermittent malaria cases from personnel deployed in Afghanistan as part of Australia’s contribution to Operation Slipper contributed to elevated case numbers in 2008 and 2010. Other malarious regions to which ADF personnel deployed and from which cases were reported include Timor Leste, until 2013, during a period of dramatic decline in country case numbers,^17,18^ and northeastern Africa, with a small contingent present from 2011.^19^ The majority species reported following infection from both Papua New Guinea and Afghanistan was *P. vivax* (**Supplementary Figure 1**), which reflects the capacity for hypnozoite-based relapse, as well as regional species prevalence.^20^ In comparison, malaria among U.S. service personnel has most commonly been acquired within Africa, with the majority of cases caused by *P. falciparum*.^14,15^ Similar to the ADF, the U.S. Armed Forces reported a reduced number of malaria cases in 2021 and 2022, attributable to the progressive withdrawal of personnel from Afghanistan and the restrictions to international travel imposed by the COVID-19 pandemic.^14,15^

A limitation to our study is the possibility that ADF CMR case notifications were incomplete, including for personnel who may have attended non-military settings for medical treatment and care. Submitted data were not always complete, and notifications without reported confirmation of malaria diagnosis were excluded, so case numbers may have been higher than those presented. ADF personnel numbers and demographics varied over the study period, although the use of 2015 denominator data was unlikely to have greatly modified results. Analysis of malaria rates against those of personnel deployed to high-risk regions over the study period would have improved clarity but was beyond the scope of this report. In 2015, 4 Royal Australian Naval (RAN) personnel were infected with *P. falciparum* malaria in Tanzania during a piracy patrol in the Indian Ocean (in AFRICOM). These cases were not reported through the CMR, affecting presentation of summary case results, including service (Navy), malaria species (*P. falciparum*), and region of malaria infection (AFRICOM).^21^

Suppressive prophylactic agents treat blood-stage malaria parasites but the 8 amino-quinolones are the only medications available to eradicate hypnozoites from the liver.^22^ Since 2000, the ADF has prescribed 30mg primaquine base daily for 14 days.^2^ CMR reporting is mandated, so most events, including relapse, are captured, although reporting indicates absent, incomplete or low-dose treatment in some instances. With aim for efficacy, ADF policy encourages advice from ADFMIDI or other infectious diseases specialists on anti-malarial management, and relapse treatment should be monitored. Some personnel experienced relapses despite documented appropriate primaquine eradication, likely due to differences in body mass and drug metabolism.^7,23^ Tafenoquine has a long half-life and efficacy against liver, blood-stage, and hypnozoite parasites, so may progressively prove useful as a weekly prophylaxis against all strains of malaria, without the need for a radical cure regimen when personnel leave a malarious area.^24^

Malaria risk to deployed military forces will continue to evolve as missions and associated geographies change. The greatest recent malaria challenge faced by the ADF was in Timor Leste beginning in 1999, with mass *P. vivax* relapse events documented following post-exposure prophylaxis and radical cure, at a time when very little endemic disease was reported.^2,25^ This emphasizes the ongoing requirement for vigilant use of malaria preventative measures and for improved diagnostics and drug regimens against hypnozoite stage parasites for regionally deployed ADF personnel.

## Figures and Tables

**Table:** Numbers of Malaria Infections in ADF Personnel, 2008–2022

	*P. vivax*	*P. falciparum*	Other Species	Total	Total	ADF Reference Population (Apr. 2015)^b^	
	No.	No.	No.	No.	%	No.	%
Total	199	27.5	152	25.4	47	37.6	27
Sex							
Male	89	12.3	68	11.4	21	16.8	30
Female	83	11.5	74	12.4	9	7.2	30
Age group, y							
<20	24	3.3	19	3.2	5	4.0	21
20-29	21	2.9	19	3.2	2	1.6	6
30-39	17	2.3	9	1.5	8	6.4	11
40-49	14	1.9	11	1.8	3	2.4	0
50+	9	1.2	7	1.2	2	1.6	3
Component							
Regular forces	8	1.1	7	1.2	1	0.8	4
Reservist	5	0.7	5	0.8	0	0.0	0
Service category							
ARA	4	0.6	4	0.7	0	0.0	5
RAN	3	0.4	3	0.5	0	0.0	2
RAAF	1	0.1	1	0.2	0	0.0	0

ADF, Australian Defence Force; *P, Plasmodium*; No., number; y, years; ARA, Australian Regular Army; RAN, Royal Australian Navy; RAAF, Royal Australian Air Force.

^a^ Count includes 1 member with a mixed *P vivax* and *P falciparum* infection.

^b^ Values may not sum due to rounding in reference document.

**Supplementary Figure 2a:**
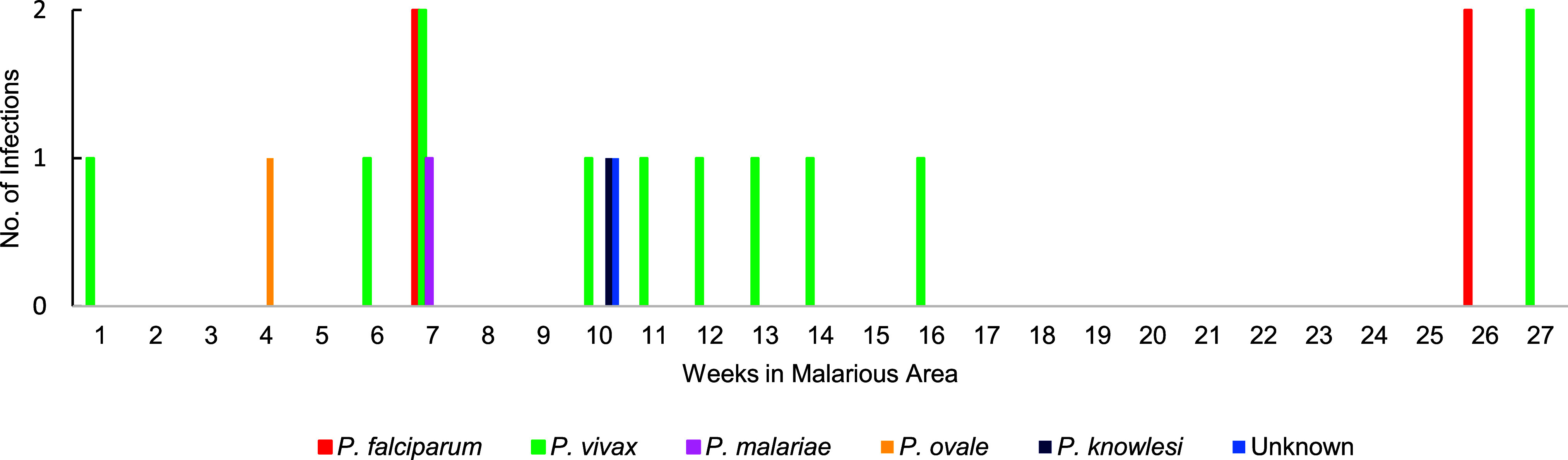
Number of Malaria Infections in ADF Personnel by Number of Weeks Since Arrival in Malarious Area

**Supplementary Figure 2b:**
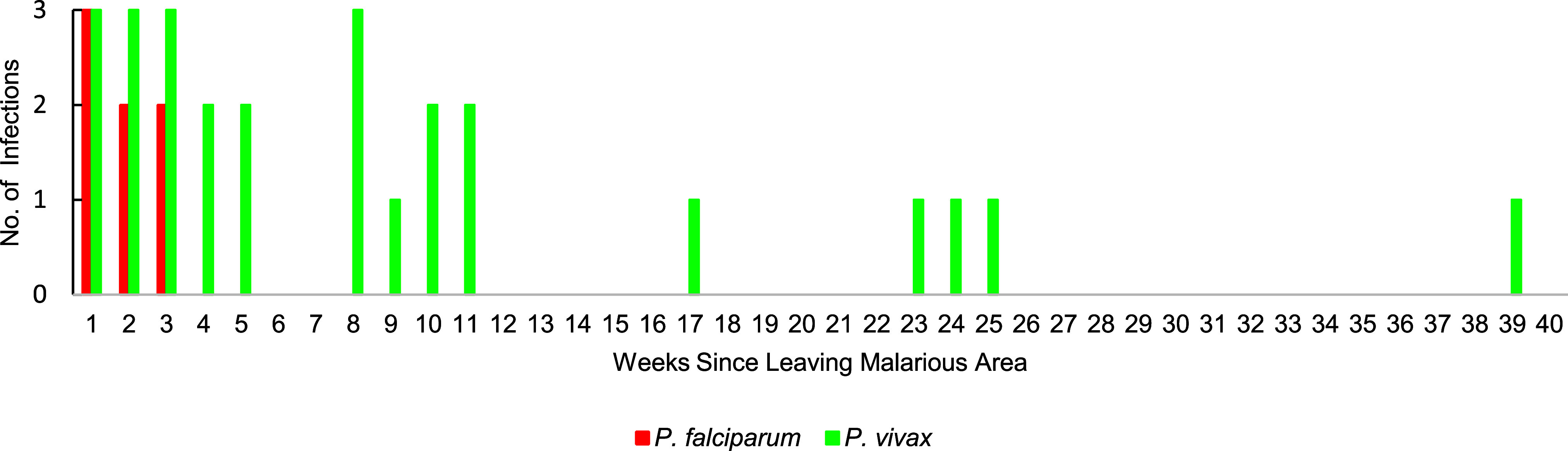
Number of Malaria Infections in ADF Personnel by Number of Weeks Since Leaving Malarious Area

## AUTHOR AFFILIATIONS

Australian Defence Force Malaria and Infectious Disease Institute, Enoggera, Queensland: CAPT Moller, LTCOL Lilley, MAJ McCallum, Dr. Shanks; University of the Sunshine Coast, Queensland, Australia: CAPT Moller; University of Queensland, School of Public Health, Brisbane, Australia: Dr. Shanks
